# Predicting lameness in dairy cattle using untargeted liquid chromatography–mass spectrometry-based metabolomics and machine learning

**DOI:** 10.3168/jds.2022-23118

**Published:** 2023-10

**Authors:** Laura V. Randall, Dong-Hyun Kim, Salah M.A. Abdelrazig, Nicola J. Bollard, Heather Hemingway-Arnold, Robert M. Hyde, Jake S. Thompson, Martin J. Green

**Affiliations:** 1 University of Nottingham, School of Veterinary Medicine and Science, Sutton Bonington Campus, Sutton Bonington, Leicestershire, LE12 5RD, United Kingdom; 2 Centre for Analytical Bioscience, Advanced Materials & Healthcare Technologies Division, School of Pharmacy, University of Nottingham, School of Pharmacy, Nottingham, NG7 2RD, United Kingdom

**Keywords:** liquid chromatography–mass spectrometry-based metabolomics, machine learning, lameness, dairy cattle

## Abstract

Lameness in dairy cattle is a highly prevalent condition that impacts on the health and welfare of dairy cows. Prompt detection and implementation of effective treatment is important for managing lameness. However, major limitations are associated with visual assessment of lameness, which is the most commonly used method to detect lameness. The aims of this study were to investigate the use of metabolomics and machine learning to develop novel methods to detect lameness. Untargeted metabolomics using liquid chromatography-mass spectrometry (LC-MS) alongside machine learning models and a stability selection method were utilized to evaluate the predictive accuracy of differences in the metabolomics profile of first-lactation dairy cows before (during the transition period) and at the time of lameness (based on visual assessment using the 0–3 scale of the Agriculture and Horticulture Development Board). Urine samples were collected from 2 cohorts of dairy heifers and stored at −86°C before analysis using LC-MS. Cohort 1 (n = 90) cows were recruited as current first-lactation cows with weekly mobility scores recorded over a 4-mo timeframe, from which newly lame and nonlame cows were identified. Cohort 2 (n = 30) cows were recruited within 3 wk before calving, and lameness events (based on mobility score) were recorded through lactation until a minimum of 70 d in milk (DIM). All cows were matched paired by DIM ± 14 d. The median DIM at lameness identification was 187.5 and 28.5 for cohort 1 and 2, respectively. The best performing machine learning models predicted lameness at the time of lameness with an accuracy of between 81 and 82%. Using stability selection, the prediction accuracy at the time of lameness was 80 to 81%. For samples collected before and after calving, the best performing machine learning model predicted lameness with an accuracy of 71 and 75%, respectively. The findings from this study demonstrate that untargeted LC-MS profiling combined with machine learning methods can be used to predict lameness as early as before calving and before observable changes in gait in first-lactation dairy cows. The methods also provide accuracies for detecting lameness at the time of observable changes in gait of up to 82%. The findings demonstrate that these methods could provide substantial advancements in the early prediction and prevention of lameness risk. Further external validation work is required to confirm these findings are generalizable; however, this study provides the basis from which future work can be conducted.

## INTRODUCTION

Lameness in dairy cattle remains a prevalent and intractable condition with profound health, welfare, and economic impacts (Huxley, 2013; Randall et al., 2019). Central to managing lameness in dairy cows is early detection to enable prompt effective treatment. This reduces lesion severity, recurrence rates, and lameness prevalence (Leach et al., 2012), whereas delay to treatment has a negative effect on recovery rates (Thomas et al., 2016). The significant contribution of previous lameness events to herd level lameness, likely due to the long-term pathological changes associated with claw horn lesions, highlights the importance of intervening early in the disease process (Newsome et al., 2016; Randall et al., 2018a). The goal therefore is accurate identification of lameness as early as possible, ideally to have the ability to predict lameness risk before the development of gait changes. Furthermore, if targeted to heifers, preventive approaches could have substantial effect on the herd over time; reducing long-term pathological changes associated with lameness and improving the health, welfare, and sustainability of the herd. Visual assessment, the current mainstay of lameness identification, has some major limitations, including resource requirements and underestimation of lameness prevalence by farmers (Whay et al., 2003; Leach et al., 2010). Additionally, visual assessment is reliant on the manifestation of pain from pathological changes that ultimately affect longer-term health and welfare. Methods to identify lame cows that are objective and could potentially identify disease risk before development of changes in mobility would offer huge advantages over visual assessment or automated sensor systems reliant on alterations in cow gait. Advanced chemical analytics such as metabolomics offer such an opportunity to achieve this through biomarker discovery.

Metabolomics using liquid chromatography-mass spectrometry (**LC-MS**) has come to the forefront as a technique for identification of disease biomarkers as well as providing unique insights into pathophysiological processes. Biomarkers are characteristics that can be objectively measured as an indicator of pathogenic processes as well as normal biological processes (Biomarkers Definitions Working Group, 2001). Metabolomics enables the comprehensive capture of metabolites that represent current phenotype and correlates with functional state. This representation of system-wide biology in real time enables the development of diagnostic tools for early detection or prediction of disease. Metabolomics-based approaches have so far been underused in livestock research (Goldansaz et al., 2017). Eckel et al. (2020) used a targeted metabolomics approach to identify 153 metabolites in urine samples of 6 severely lame and 20 nonlame cows using mass spectrometry. This preliminary study suggested differences in metabolites may be identified in lame and nonlame cows as early as precalving, which requires further validation. The transition period (3 wk before to 3 wk after calving) has been highlighted as a potentially important time as metabolic changes associated with calving may influence pathological processes occurring in the hoof that lead to lesions causing lameness; however, further work is still required to fully understand these processes and temporal associations (Randall et al., 2018b). Metabolomics may offer an opportunity to achieve this goal through investigation of metabolite biomarkers associated with lameness.

Untargeted metabolomics, not restricted to a subset of a priori chosen biomolecules, offers a comprehensive and unbiased approach for metabolite analysis (Alonso et al., 2015). The untargeted analysis of metabolites, using sensitive techniques such as LC-MS, captures rich biological information relating to disease processes. The complex multidimensional data generated using LC-MS does, however, require appropriate statistical and computational techniques to translate the captured information for use in clinical application (Lee and Hu, 2019). Machine learning methods can be used to analyze multidimensional data sets, which enables the predictive power of data generated by advanced chemical analytical techniques to be optimized.

The aims of the current study were to use machine learning methods to identify differences in the urinary metabolomic profiles of lame and nonlame dairy cows and to evaluate the predictive accuracy of the metabolomic differences in first-lactation cows before (during the transition period) and at the time of lameness. Therefore, the primary objectives of this study were to (1) evaluate predictive accuracy of the metabolomic profile of lame and nonlame dairy cows at the time of lameness during first lactation, and (2) evaluate the predictive accuracy of the metabolomics profile of lame and nonlame heifers during the transition period as they enter first lactation.

## MATERIALS AND METHODS

Ethical approval for this case-control study was granted from the University of Nottingham Committee for Animal Research and Ethics (Reference No. 3120 200220 and 3132 200309). First-lactation Holstein dairy cows were recruited in 2 separate cohorts from one dairy herd based at the Centre for Dairy Science Innovation, University of Nottingham. The herd is a 300-cow research dairy herd producing milk commercially. Cows were housed continuously with sand-bedded cubicles and slatted flooring. Milking was via automatic (robotic) milking stations (Lely Astronaut A3; Lely UK Ltd.). Cows were fed a TMR, offered ad libitum, and a concentrate feed offered during milking in the automated milking system that was according to milk yield (0.45 kg/kg milk yield above 32 kg/d, up to maximum of 12 kg/d or 3 kg per automatic milking station visit).

### Heifer Cohorts and Mobility Scoring

Two cohorts of heifers were recruited. Cohort 1 were recruited as current first-lactation cows with weekly mobility scores being recorded over a 4-mo timeframe from which lame and nonlame cows (based on mobility score) were defined. Cohort 2 were recruited within 3 wk before calving, and lameness events (based on mobility score) were recorded through their first lactation until a minimum of 70 DIM; lame and nonlame cows were defined during this period. All mobility scoring was conducted using the Agriculture and Horticulture Development Board 0 to 3 scale (AHDB, 2022) scoring system by one of 3 trained mobility scorers. Calibration sessions were conducted on multiple occasions during the study periods and Gwet's AC_1_ calculated from the calibration mobility score data (Gwet, 2014). Minimum cohort sizes were based on published pilot data in the same species using the same similar statistical analytical framework to analyze mass spectral data (He et al., 2022). A brief description of each cohort is outlined below including timings of sampling and definitions of lameness;

#### Cohort 1

A total of 175 first-lactation dairy cows were mobility scored weekly between July 24 and November 19, 2020. All first-lactation cows in the herd were eligible for inclusion, apart from those having undergone surgery or any treatment with antimicrobials or anti-inflammatories in the current lactation. On identification of a new case of lameness, defined as a mobility score of <2 for at least 2 wk before being scored ≥2 for a minimum of 2 consecutive weeks, urine samples were collected (at lameness) and stored according to the protocols described below. For each lame cow, match paired (±14 DIM) nonlame cows were identified and sampled in the same manner. A nonlame cow was defined as having at least 2 consecutive weeks of score <2 before sampling followed by at least 2 consecutive weekly scores <2. Once the sample collection phase of the study had been completed (so that mobility scores were available to enable confirmation that the criteria for lame and nonlame had been met by a total of 90 cows), samples were selected and transported for metabolomics analysis.

#### Cohort 2

A total of 67 dairy heifers (entering their first lactation) were recruited precalving during August 7, 2020, to March 11, 2021. Mobility scoring was conducted weekly from up to 3 wk before calving to a minimum of 70 DIM. Urine samples were collected, according to protocols described below, from all heifers within 3 wk before calving and within 3 wk after calving. Samples were also collected at the time of first case of lameness from lame cows and match paired nonlame control cows. Lameness was defined as above, with nonlame cows defined as having no 2 consecutive weekly score ≥2 within 70 DIM. Heifers undergoing surgery or treatment with anti-inflammatories or antimicrobials during the study period were excluded. Once the sample collection phase of the study had been completed (so that mobility scores were available up to a minimum of 70 DIM to confirm that the criteria for lame and nonlame had been met by a total of 30 heifers), samples were selected and transported for metabolomics analysis.

### Sample Collection and Handling Protocols

Urine samples were collected via free-flow catch and gloves were worn at all times during sample collection and handling. Urine is widely used in metabolomics studies due to the minimally invasive nature by which it can be collected and its usefulness in providing insights into the metabolome at a systemic level (Fernández-Peralbo and Luque de Castro, 2012). Warm water and paper towels were used to wash the exterior vulva and surrounding area before urine collection. Samples were collected into universal containers, transferred to cryovials (1–2 mL) and immediately frozen in liquid nitrogen. Samples were stored at −86°C (Upright Ultra-Low Temperature Freezer, Thermo Fisher Scientific, Hemel Hempstead, UK) and transported on dry ice to the Centre for Analytical Bioscience, University of Nottingham.

### Untargeted Metabolomics Using LC-MS

Samples from cohort 1 (at lameness) were processed together in one analytical run and samples from cohort 3 (precalving, postcalving, and at lameness) were processed together in one separate analytical run.

### Chemicals and Reagents for LC-MS

Deionized water was prepared using Milli-Q water purification system (Millipore). Acetonitrile and methanol were LC-MS grade and obtained from VWR (West Sussex, UK) and Fisher Scientific (Loughborough, UK), respectively. All analytical standards and chemicals including ammonium carbonate were purchased from either Fisher Scientific (Loughborough, UK) or Sigma-Aldrich (Gillingham, UK) unless otherwise stated. For metabolite identification, 5 mixtures containing 268 authentic standards were prepared in methanol:water (1:1) and were used for the identification of the metabolites as detailed elsewhere (Abdelrazig et al., 2020).

### Sample Preparation for LC-MS

The preparation of urine samples was conducted as described by Want et al. (2010). In brief, 60 µL of urine samples were thawed on ice and centrifuged at 10,000 × *g* at 4°C for 10 min. Fifty microliters of the supernatant of the urine samples were transferred into HPLC vials and diluted with 100 µL of deionized water. The urine samples were then vortexed for 30 s and analyzed with LC-MS. Reagent blank was prepared following the same procedure excluding the sample. For metabolomics analysis, a pooled quality control (QC) sample was prepared by mixing 20 µL from each urine sample and vortexed for 30 s.

### Analytical Methodologies

The LC-MS for metabolite profiling and LC-MS/MS for metabolite identification were performed using a Q-Exactive Plus mass spectrometer equipped with Dionex U3000 UHPLC system (Thermo Fisher Scientific, Hemel Hempstead, UK). A ZIC-*p*HILIC column (4.6 × 150 mm, 5-µm particle size, Merck SeQuant, Watford, UK) was used for the chromatographic separation of the metabolites in the samples, with a flow rate of 300 µL min^−1^ at 45°C for 15 min. The samples were maintained at 4°C and the injection volume was 5 µL. The gradient started with 20% A (20 m*M* ammonium carbonate in water) and 80% of B (acetonitrile) and increased to 95% A over 8 min, before returning the composition to the initial conditions in 2 min at 400 µL/min and the column was re-equilibrated for 5 min at 300 µL/min (15 min total time). The MS was operated in electrospray ionization positive and negative (**ESI+** and **ESI−**, respectively) switching acquisition mode for LC-MS profiling and in data-dependent MS/MS for identification of metabolites (QC sample). Spray voltage was 4.5 kV (ESI+) and 3.5 kV (ESI−), capillary voltage was 20 V (ESI+) and −15 V (ESI−) and the sheath, auxiliary and sweep gas flow rates were 40, 5, and 1 (arbitrary unit), respectively. Capillary and heater temperatures were 275 and 150°C, respectively. A resolution of 70,000 from *m/z* 70 to 1,050 was used to acquire data for LC-MS profiling. A resolution of 17,500 and a stepped normalized collision energy of 20, 30, and 40 were used for the identification of metabolites using data-dependent MS/MS.

### LC-MS Untargeted Metabolomics Analysis and Metabolite Identification

Urine samples, standard mixtures (n = 5), blanks (n = 3), and QC sample were analyzed in a single analytical run for each cohort. The pooled QC sample were analyzed at the beginning of analysis (n = 6) to equilibrate the column. The urine samples were randomized and the QC sample was analyzed after every 9 samples to test the performance of the LC-MS analytical system.

### Data Analysis and Metabolite Identification

The acquired raw LC-MS data sets were processed with Compound Discoverer 3.1 SP1 software (Thermo Fisher Scientific, Hemel Hempstead, UK). The LC-MS analytical performance was assessed using the pooled QC approach by unsupervised principal component analysis (**PCA**, Simca P +164; Sartorius Stedim Data Analytics AB, Umea, Sweden; Gika et al., 2007; Want et al., 2010). In brief, the data sets were log-transformed using Compound Discoverer and imported to Simca P for multivariate analysis including PCA. The imported data sets were mean-centered and Pareto scaled. The robustness of the generated PCA model for metabolomics analysis was evaluated using cross-validation by monitoring the fitness of model (R^2^X) value.

Subsequent analysis of each of the LC-MS profiles (mass ions) generated for each cohort (cohort 1: samples collected at lameness; cohort 2: at lameness, precalving, and postcalving) was conducted using R (R Core Team, 2022) with lameness (lame or nonlame) as a binary outcome. A suite of machine learning models including support vector machine (**SVM**; Cortes and Vapnik, 1995), elastic net regression (Zou and Hastie, 2005), partial least squares regression (Wold et al., 2001), Random forest (Breiman, 2001) and multivariate adaptive regression splines (**MARS**; Friedman, 1991) were utilized within the caret package (Kuhn, 2022). Data were normalized to total ion count and standardized. Recursive feature elimination is a sequential method of model construction with reducing numbers of variables; variables are removed in order of importance until an optimal model is achieved based on the best cross-validation performance with the smallest number of variables (Kuhn and Johnson, 2013). Recursive feature elimination was used for each algorithm to select the smallest number of mass ions that provided the best performance accuracy in terms of classifying cows as lame or nonlame. The number of features that could be selected were between 2 and 200. Mean prediction accuracy and performance metrics including sensitivity, specificity, positive predictive values (**PPV**) and negative predictive values (**NPV**) were assessed using leave-one-out cross-validation. The best performing models were identified based on mean prediction accuracy. In addition, stability selection was conducted using the stabilizer package (Hyde et al., 2022a) to prevent overfitting and identify the minimum number of variables that classify cows as lame or nonlame. This approach is based on the principles described by Meinshausen and Bühlmann (2010), whereby repeated subsampling (bootstrapping) is used to identify covariates most frequently selected across bootstrap samples. A threshold for selection frequency for variable inclusion is identified by comparing the proportion of covariates selected using the observed data versus a data set in which the outcome is randomly permutated (Hyde et al., 2022b). Model triangulation, to select variables by multiple modeling approaches, further reduces the probability of selecting false positive variables (Lima et al., 2021). We conducted model triangulation using 3 regularized regression models based on (1) a modified Bayesian information criterion (Zhang and Siegmund, 2007), (2) the minimax convex penalty (Zhang, 2010), and (3) least absolute shrinkage and selection operator regression (Friedman et al., 2010). Following stability analysis and model triangulation, performance metrics were calculated for the final selected variables using leave-one-out cross-validation.

## RESULTS

### Descriptive Statistics

#### Cohort 1

Samples from a total of 90 heifers [45 lame and 45 nonlame (control)] were analyzed using LC-MS. Body condition score ranged between 2.5 to 4, and DIM ranged between 2 to 318. The median DIM at lameness identification was 187.5 d (Table 1). All matched pairs met the criteria of being within 14 DIM. All lame cows were scored 2 for a minimum of 2 consecutive weeks, apart from one cow in cohort 2 with a single score 3 (followed by a 2 the subsequent week).

**Table 1:** Descriptive statistics for DIM/days before calving when sampled relating to 2 cohorts of dairy heifers sampled for urine (n = 172 samples) for metabolomics analysis

Data set^1^	DIM/days precalving when sampled		
	All	Lame	Nonlame (control)
Cohort 1; at lameness (n = 90)			
Range	2–318	2–318	11–313
Median	187.5	187	188
Interquartile range	50.25–233.75	50–234	53–233
Cohort 2; at lameness (n = 26)			
Range	7–76	7–76	7–64
Median	28.5	29	28
Interquartile range	20–51.75	22–56	20–51
Cohort 2; precalving (n = 28)			
Range	1–15	1–13	3–15
Median	5.5	4.5	6.5
Interquartile range	4–7.75	3.00–6.75	4.2–10.25
Cohort 2; postcalving (n = 28)			
Range	0–20	0–15	1–20
Median	4	3.50	5.00
Interquartile range	3–7.5	3.00–5.75	3.00–3.50

1 Cohort 1: Urine samples collected from a total of 90 heifers [45 lame and 45 nonlame (control)]. Cohort 2: Urine samples collected from a total of 30 heifers (15 lame and 15 nonlame). At lameness: samples collected at the time of lameness. Precalving: samples collected within 3 wk precalving. Postcalving: samples collected within 3 wk postcalving.

#### Cohort 2

Samples from a total of 30 heifers [15 lame and 15 nonlame (control); sample numbers at each stage differed due to small numbers of match paired samples not being available] were analyzed using LC-MS. The minimum BCS at any time point was 3 and the maximum was 4. Days in milk ranged from 7 to 76 at the time of lameness, whereas samples collected precalving were from heifers between 1 and 15 d before calving, and samples collected postcalving were from heifers 0 to 20 d after calving (Table 1). The median DIM at lameness identification was 28.5 d and all matched pairs met the criteria of being within 14 DIM (Table 1). The Gwet's AC1 coefficient (SE) was 0.60 (0.07) for the 3 mobility scorers.

### Untargeted LC-MS Metabolomics

The LC-MS profiles generated 2,680 mass ions from the samples collected at the time of lameness for cohort 1. For cohort 2, the LC-MS profiles generated 4,383 mass ions for samples collected precalving, postcalving, and at the time of lameness. Each of these LC-MS data sets were subsequently analyzed with all mass ions included in the analysis. Pooled QC samples were clustered toward the center of the PCA scores plot with all samples indicating good stability and validity of LC-HRMS analytical performance for cohort 1 and cohort 2.

### Performance Metrics

The performance metrics for all data sets and data analytical approaches are summarized in Table 2. The mean prediction accuracy of the best performing machine learning model (SVM) for cohort 1, with samples taken at the time of lameness, was 82.2% with 10 variables selected. For cohort 2, the highest prediction accuracy from the best performing machine learning model (MARS) was 80.8% with 2 variables selected in the model for samples taken at the time of lameness. At the time of lameness, sensitivity of between 0.80 (cohort 1) and 0.92 (cohort 2) were achieved, and specificity of between 0.84 and 0.69 for cohort 1 and 2, respectively, while PPV of between 0.84 (cohort 1) to 0.75 (cohort 2) and NPV 0.81 (cohort 1) to 0.9 (Cohort 2) were achieved. Using stability selection, the prediction accuracy of selected metabolites was 79.9% (5 variables selected) and 80.8% (1 variable selected) for samples taken at the time of lameness for cohort 1 and 2, respectively. While the sensitivity was 0.76 to 0.84, specificity was 0.77 to 0.82, PPV was 0.79 to 0.81, and NPV 0.77 to 0.83 across the 2 cohorts using these methods (samples collected at the time of lameness and performance metrics calculated from metabolites selected using stability selection). For precalving samples (median sampling time was 5.5 d precalving), the best performing machine learning model (random forest) provided a prediction accuracy of 71.4% with 15 variables selected in the model, for lameness occurring in the following lactation. Sensitivity, specificity, PPV, and NPV were 0.5, 0.71, 0.64, and 0.59 respectively. For postcalving samples (median sampling time was 4 d postcalving) the highest prediction accuracy from the best performing machine learning model (random forest) was 75.0% with 15 variables selected. Sensitivity, specificity, PPV, and NPV were 0.71, 0.50, 0.59, and 0.63, respectively. For both data sets from samples collected within the transition period (3 wk precalving to 3 wk postcalving) no variables were selected using stability selection and therefore no predictions were made using this method.

**Table 2:** Performance metrics [mean prediction accuracy (point estimate), sensitivity, specificity, PPV, NPV] for mass ions selected by machine learning (ML) models and stability selection from liquid chromatography-mass spectrometry data generated using untargeted metabolomics on urine samples from dairy heifers^1^

Data set^2^	Best performing ML model	No. variables selected	Mean accuracy (%)	Sensitivity	Specificity	PPV	NPV
Cohort 1							
At lameness (n = 90)	SVM^3^	10	82.2	0.80	0.84	0.84	0.81
	Stability analysis	5	79.9	0.76	0.82	0.81	0.77
Cohort 2							
At lameness (n = 26)	MARS^4^	2	80.8	0.92	0.69	0.75	0.90
	Stability selection	1	80.8	0.84	0.77	0.79	0.83
Precalving (n = 28)	Random forest	15	71.4	0.50	0.71	0.64	0.59
	Stability selection	None	NA^5^	NA	NA	NA	NA
Postcalving (n = 28)	Random forest	15	75.0	0.71	0.50	0.59	0.63
	Stability selection	None	NA	NA	NA	NA	NA

1 Best preforming models were selected based on having the highest mean accuracy of all algorithms evaluated. PPV = positive predictive value; NPV = negative predictive value.

2 Cohort 1: Urine samples collected from a total of 90 heifers [45 lame and 45 nonlame (control)]. Cohort 2: Urine samples collected from a total of 30 heifers (15 lame and 15 nonlame). At lameness: samples collected at the time of lameness. Precalving: samples collected within 3 wk precalving. Postcalving: samples collected within 3 wk postcalving.

3 SVM = support vector machine.

4 MARS = multivariate adaptive regression splines.

5 NA = not applicable.

### Triangulation of Top-Ranking Mass Ions and Selection Using Stability Selection Method

Of the top 10 ranking mass ions output from the best performing machine learning (**ML**) algorithm and the top 5 ranking mass ions selected by the stability selection method, between one and 4 mass ions were found to be the same across both methods (ML algorithm and stability selection) for each data set. These mass ions have a higher certainty of being correctly identified as being predictive for lameness for that particular data set. For samples collected at the time of lameness, there was one mass ion that was top ranking in the best performing ML model (SVM) and was also selected using stability selection for cohort 1. Likewise, there was one mass ion that was top ranking in the best performing ML model (MARS) and selected using stability selection for cohort 2. For samples collected precalving (cohort 2) there were 3 mass ions that were top ranking in the best performing ML model (random forest) that were also selected using stability selection. For samples collected postcalving there were 4 mass ions that were top ranking (random forest) and selected using stability selection. Using stability selection 5 mass ions were selected in the model for samples collected at the time of lameness in cohort 1 and one mass ion for samples collected at the time of lameness in cohort 2. For samples collected pre- and postcalving no mass ions were selected suggesting more uncertainty in these being predictive of lameness versus those selected at the time of lameness.

## DISCUSSION

This study is the first to report on the use of untargeted LC-MS metabolomics combined with machine learning algorithms to determine the prediction accuracy of urinary metabolomics profiles for lameness in dairy cows. Furthermore, the differences in the urinary metabolomics profiles were evaluated at time points both around the time of calving (transition period) and at the time of lameness detection, to determine whether the differences in the metabolomics profiles could be predictive for lameness at an early stage before changes in gait, as well as once gait changes are visible. At the time of lameness, prediction accuracies of up to 82% were achieved using these methods. Around the time of calving, prediction accuracies of 71 to 75% were achieved, before changes in gait were being observed. The reference standard outcome for lameness, against which these accuracies were calculated, was mobility score (visual assessment of gait). As it is well acknowledged that mobility scoring is a subjective measure with high inter-rater variability and poorer accuracy for detecting milder lameness, this may contribute to lower prediction accuracies of the mass ions selected by algorithms due to misclassification of the outcome in the first place. It is possible that the metabolomics profile was in fact better at detecting lameness than by mobility scoring. Furthermore, these prediction accuracies may be increased with the use of targeted metabolomics compared with untargeted metabolomics, once target metabolites have been established. Future studies may also be able to improve on the accuracies reported here with the addition of lesion data to define lameness outcomes. It is likely that the cause of lameness, and whether this is infectious or noninfectious, will affect the metabolome of lame cows.

One of the key potential uses for metabolomics is in diagnostic applications and this has been evidenced across multiple diseases in humans, including cancer, diabetes and cardiovascular diseases (Gowda et al., 2008). Studies evaluating the use of metabolomics to develop diagnostic biomarker panels in dairy cattle have been reported in a small number of diseases including mastitis and ketosis (Hu et al., 2021; Zhang et al., 2021). For example, Zhang et al. (2021) reported that the area under the curve from receiver operating characteristics curve of a small 6 biomarker panel for predicting ketosis at −8 and −4 wk were 0.98 and 0.99, respectively, demonstrating the potential predictive capabilities of metabolomics for livestock diseases. In terms of lameness, one pilot study has reported an area under the curve of 0.98 (95% CI: 0.76–1) and 0.99 (95% CI: 0.94–1) at −4 and −8 wk postpartum, respectively (Eckel et al., 2020). This was achieved using the top 5 biomarkers identified in the urine of severely lame (n = 6) compared with control (n = 20) cows utilizing a targeted LC-MS metabolomics; however, no other performance metrics such as accuracy were reported, and authors highlight the preliminary nature of these results that require further external validation. However, the results do support the findings of the current study, in which it was found that mass ions predictive of lameness can be detected as early as before calving (for the lactation in which the cows become lame). In the current study, prediction accuracies up to 75% were achieved using samples collected in the transition period (3 wk pre- to 3 wk postcalving). For samples collected precalving, this was a minimum of 8 d and a maximum of 22 d before the detection of lameness using visual assessment. These results demonstrate the potential for using these methods to develop tools for early warning or prediction of lameness before any visible changes in gait.

The precalving and postcalving samples collected during the transition period were used in the current study as the transition period has been identified as an important time period for influencing lameness risk due to hormonal and metabolic changes taking place (Webster, 2001; Tarlton et al., 2002). Additionally, subacute inflammation occurring during transition is thought to play a role influencing subsequent health status (Bradford et al., 2015). The findings of the current study suggest that metabolic alterations are indeed occurring during the transition period that increase the risk of lameness; metabolomics profiles during this time period were predictive of lameness later in lactation. Eckel et al. (2020) and Dervishi et al. (2020) reported findings from studies of the same small cohort of severely lame and healthy dairy cows that identified metabolic alterations of lame cows up to 8 wk before calving as well as during the transition period, although it should be noted all lameness cases occurred within 2 wk postcalving (i.e., during the transition period).

Prediction accuracies up to 82% were achieved for both mildly and newly lame cows using samples collected at the time of lameness (identified visually) in addition to those collected before lameness. This is important when considering the benefits of using these techniques for early diagnosis or prediction of lameness. The lameness definitions used in this study meant samples were from the first new lameness event scored as mild to moderate, or a mild to moderate lameness event that was not preceded by lameness within a minimum of 2 wk in first-lactation cows. These definitions and inclusion criteria meant that potential effects of lameness history were either eliminated or reduced considerably. In the study reported by Eckel et al. (2020), the severity of lameness was much higher with a lameness definition of cows scored 4 or 5 on a 1-to-5 scale. Findings therefore may not be relevant for milder to moderate lameness, which is most commonly observed in dairy herds. In a study reported by He et al. (2022), conducted at the same herd as the current study and also utilizing untargeted metabolomics, a lameness definition of score ≥2 (0–3 scale; AHDB, 2020) was used, encompassing the milder lameness definition. Prediction accuracies of 95 to 100% were achieved using milk samples (dried milk spots) collected on Whatman FTA DMPK cards (Qiagen). The current study focused on the inclusion of milder lameness in dairy heifers, as this is the population in which prevention and treatment strategies will have most effect, particularly with early detection and prompt effective treatment (Randall et al., 2018a; Pedersen and Wilson, 2021). Studies have demonstrated that early lameness interventions improve treatment outcomes (Leach et al., 2012) and that delays to treatment result in the converse (Groenevelt et al., 2014; Thomas et al., 2016). As reliability of lameness classification is lower for milder lameness versus severe lameness and single gait scores can contribute to misclassification (Eriksson et al., 2020), 2 consecutive weekly scores were used to classify lameness outcomes in the current study. Prediction accuracies were lower than those reported by He et al. (2022), which may be due to differences in the biofluid (milk vs. urine) or chemical analytical methods employed.

Metabolite identification was not a focus of the current study; however, the methods adopted, including triangulation of top-ranking mass ions from multiple analytical approaches and use of the stability selection method, provides greater confidence in the ability to correctly identify top-ranking or selected mass ions that are predictive of the disease outcome compared with more traditional approaches to analyzing data from LC-MS, such as PCA or orthogonal projections to latent structures discriminant analysis. The usefulness in determining a metabolic signature that is predictive of lameness is for (1) development of diagnostic tools and (2) metabolite identification and pathway analysis to elucidate mechanistic pathways associated with those metabolites. One of the major challenges with this later approach is ensuring the “top-ranking” or “selected” metabolites are truly associated with the lameness outcome. This is particularly pertinent with untargeted metabolomics studies where there is no a priori subset of metabolites. Untargeted metabolomics provides an opportunity to uncover unknown pathways and important metabolites, which is a major benefit of this approach at these exploratory stages. However, the data analysis must be appropriate for the complex LC-MS data that this highly sensitive approach generates. Model triangulation and selection stability are methods reported to improve likelihood of selecting the correct variables from multidimensional data sets, and furthermore, implementation of a stability threshold to identify the correct covariates has been demonstrated to out-perform conventional stepwise methods of implementing machine learning models (Lewis et al., 2021; Lima et al., 2021). Further work is required to confirm the identity of mass ions identified in this study as well as to demonstrate the generalizability through external validation studies. However, these findings inform the basis from which this confirmatory and validatory work can be conducted and if successful, these techniques could then provide the building blocks for the development of commercial tools for the early prediction and identification of lameness. These techniques could also be applied for the investigation or other diseases and conditions. Additionally, pathway analysis of validated predictive metabolites would provide important insights into the pathogenesis of lameness. This would contribute valuable information to understanding the pathological pathways at different time points both during the transition period as well as closer to the time of lameness becoming visibly apparent. Ultimately, this would aid in developing strategies to improve the prevention or management of lameness in dairy cattle.

## CONCLUSIONS

The findings from this study demonstrate that the use of untargeted LC-MS-based metabolomics alongside machine learning and a stability selection method can predict lameness risk in dairy cows, from as early on as before calving. Prediction accuracies up to 82% were achieved using these methods at the time of lameness. Further work to demonstrate the generalizability of findings, through external validation studies is required, as well as confirmation of metabolite identification. Using these techniques to develop methods for the early and accurate prediction of lameness risk provides a huge opportunity to build tools to enable the improvement of lameness management and reduce lameness in dairy herds.
